# The Vitamin D Receptor in Osteoblast-Lineage Cells Is Essential for the Proresorptive Activity of 1α,25(OH)_2_D_3_ In Vivo

**DOI:** 10.1210/endocr/bqaa178

**Published:** 2020-09-28

**Authors:** Tomoki Mori, Kanji Horibe, Masanori Koide, Shunsuke Uehara, Yoko Yamamoto, Shigeaki Kato, Hisataka Yasuda, Naoyuki Takahashi, Nobuyuki Udagawa, Yuko Nakamichi

**Affiliations:** 1 Graduate School of Oral Medicine, Matsumoto Dental University, Shiojiri, Nagano, Japan; 2 Department of Oral Histology, Matsumoto Dental University, Shiojiri, Nagano, Japan; 3 Institute for Oral Science, Matsumoto Dental University, Shiojiri, Nagano, Japan; 4 Department of Biochemistry, Matsumoto Dental University, Shiojiri, Nagano, Japan; 5 Department of Surgical Oncology, The University of Tokyo, Tokyo, Japan; 6 Research Institute of Innovative Medicine, Tokiwa Foundation, Iwaki, Fukushima, Japan; 7 Department of Basic Pathology, Fukushima Medical University, Fukushima, Japan; 8 Nagahama Institute for Biochemical Science, Oriental Yeast Co., Ltd., Nagahama, Shiga, Japan

**Keywords:** Proresorptive action, hypercalcemia, toxic action, osteoblast-lineage cells, VDR, Ob-VDR-cKO mice

## Abstract

We previously reported that daily administration of a pharmacological dose of eldecalcitol, an analog of 1α,25-dihydroxyvitamin D_3_ [1α,25(OH)_2_D_3_], increased bone mass by suppressing bone resorption. These antiresorptive effects were found to be mediated by the vitamin D receptor (VDR) in osteoblast-lineage cells. Using osteoblast-lineage-specific VDR conditional knockout (Ob-VDR-cKO) mice, we examined whether proresorptive activity induced by the high-dose 1α,25(OH)_2_D_3_ was also mediated by VDR in osteoblast-lineage cells. Administration of 1α,25(OH)_2_D_3_ (5 μg/kg body weight/day) to wild-type mice for 4 days increased the number of osteoclasts in bone and serum concentrations of C-terminal crosslinked telopeptide of type I collagen (CTX-I, a bone resorption marker). The stimulation of bone resorption was concomitant with the increase in serum calcium (Ca) and fibroblast growth factor 23 (FGF23) levels, and decrease in body weight. This suggests that a toxic dose of 1α,25(OH)_2_D_3_ can induce bone resorption and hypercalcemia. In contrast, pretreatment of wild-type mice with neutralizing anti-receptor activator of NF-κB ligand (RANKL) antibody inhibited the 1α,25(OH)_2_D_3_-induced increase of osteoclast numbers in bone, and increase of CTX-I, Ca, and FGF23 levels in serum. The pretreatment with anti-RANKL antibody also inhibited the 1α,25(OH)_2_D_3_-induced decrease in body weight. Consistent with observations in mice conditioned with anti-RANKL antibody, the high-dose administration of 1α,25(OH)_2_D_3_ to Ob-VDR-cKO mice failed to significantly increase bone osteoclast numbers, serum CTX-I, Ca, or FGF23 levels, and failed to reduce the body weight. Taken together, this study demonstrated that the proresorptive, hypercalcemic, and toxic actions of high-dose 1α,25(OH)_2_D_3_ are mediated by VDR in osteoblast-lineage cells.

Vitamin D_3_ is an antirachitic factor produced by sunlight ultraviolet irradiation of 7-dehydrocholesterol in the skin (1, 2). In addition, vitamin D_3_ is supplied via food intake (1, 2). Severe vitamin D deficiency causes hypocalcemia and insufficient calcification of osteoid, which result in rickets and osteomalacia (1, 2). Vitamin D_3_ is hydroxylated at the C-25 position and converted to 25-hydroxyvitamin D_3_ [25(OH)D_3_] by the hepatic cytochrome P450 enzymes CYP2R1 and CYP27A1 (3, 4). The 25-hydroxylation of vitamin D_3_ in the liver is rapid and not regulated. 25(OH)D_3_ is then hydroxylated at the C-1α position by the renal 25(OH)D-1α-hydroxylase (CYP27B1), resulting in the generation of the biologically active form of vitamin D_3_, 1,25-dihydroxyvitamin D_3_ [1α,25(OH)_2_D_3_] (5). The expression of 1α-hydroxylase is negatively regulated by 1α,25(OH)_2_D_3_ itself, high dietary calcium (Ca), high dietary phosphorus (as “phosphate”), and fibroblast growth factor 23 (FGF23), a phosphaturic bone-derived hormone (6-10), and positively regulated by low dietary Ca and parathyroid hormone (6, 9-11). 1α,25(OH)_2_D_3_ produced in the kidneys is transported to the target organs via blood circulation (2). It then binds to the vitamin D receptor (VDR) and upregulates serum Ca levels through intestinal Ca absorption, renal tubular reabsorption of Ca, and bone resorption (2). Thus, 1α,25(OH)_2_D_3_ is widely recognized as a calcitropic hormone due to its tightly controlled renal production, blood-mediated delivery to the target tissues, and regulated actions through present VDR (2).

1α,25(OH)_2_D_3_ stimulates osteoclastic bone resorption in rat bone organ cultures (12). It can also stimulate osteoclast formation in coculture of mouse osteoblastic cells and hematopoietic cells (13). Osteoblastic cells express receptor activator of NF-κB ligand (RANKL) and osteoprotegerin (OPG), thereby regulating the generation of osteoclasts. 1α,25(OH)_2_D_3_ increases the RANKL/OPG ratio by upregulating RANKL and downregulating OPG gene transcription via VDR (14-16).

On the contrary, long-term treatment with pharmacological doses of active vitamin D_3_ drugs, as well as sustained overexpression of VDR in mature osteoblast-lineage cells, has been demonstrated to increase bone mass by suppressing bone resorption (17-25). Due to this efficacy, 3 vitamin D_3_ analogs, 1α(OH)D_3_ (alfacalcidol), 1α,25(OH)_2_D_3_ (calcitriol), and 1α,25(OH)_2_-2β-(3-hydroxy-propyloxy) D_3_ (eldecalcitol), have been approved for the treatment of osteoporosis in Japan (17, 20, 23). Alfacalcidol undergoes 25-hydroxylation in the liver and acts as 1α,25(OH)_2_D_3_, whereas eldecalcitol functions as a hormonally active vitamin D_3_ by directly binding VDR (26, 27). Alfacalcidol is shown to inhibit bone resorption and stimulate formation in an ovariectomized rat model of osteoporosis (18). Transgenic overexpression of VDR in mature osteoblast-lineage cells also decreased bone resorption and increased formation under physiological conditions (24, 25). The inhibition of bone resorption by alfacalcidol was suggested to be due to decreasing the pool of osteoclast precursors (19). In Phase 2 clinical studies on osteoporosis in 2005, treatment with eldecalcitol for 12 months significantly increased bone mineral density and reduced urinary levels of N-terminal crosslinked telopeptide of type I collagen, a bone resorption marker, in a dose-dependent manner (28). Therefore, it has been confirmed both experimentally and clinically that long-term treatment with pharmacological doses of active vitamin D_3_ analogs suppresses bone resorption and results in a net increase in bone mass.

To evaluate the effects of eldecalcitol on bone resorption and formation, we administered a pharmacological dose of eldecalcitol (50 ng/kg of body weight/day) daily for 4 weeks to C57BL/6 male mice aged 9 weeks (29). We confirmed that eldecalcitol administration increased bone mass by suppressing bone resorption via downregulation of the RANKL/OPG ratio in bone tissues without affecting the number of osteoclast precursors (29). To clarify which type of VDR-expressing cells preferentially regulated the eldecalcitol-induced increase in bone mass, we generated osteoblast-lineage (osteoblast and osteocyte)-specific VDR conditional knockout [Ob-VDR-cKO, osterix (Osx)-Cre^Tg/0^; VDR^fl/fl^] and osteoclast-specific VDR-cKO [Ocl-VDR-cKO, cathepsin (Ctsk)^Cre/+^; VDR^fl/fl^] mice (30). Administration of eldecalcitol for 4 weeks neither suppressed bone resorption nor increased bone mass in Ob-VDR-cKO mice (30). In contrast, it suppressed bone resorption and subsequently increased bone mass in Ocl-VDR-cKO mice and control mice (30). These results suggested that VDR in osteoblast-lineage cells, but not enterocytes, renal cells, or osteoclasts, primarily mediates the eldecalcitol and other bioactive vitamin D-induced increase in bone mass (14, 15, 24, 25, 29, 30).

In the present study, we examined (i) whether administration of large amounts of 1α,25(OH)_2_D_3_ increases bone resorption in vivo, and if so, (ii) whether the proresorptive effects are mediated by VDR in osteoblast-lineage cells. High-dose administration of 1α,25(OH)_2_D_3_ to normal mice stimulated osteoclastic bone resorption with increasing serum Ca levels, intact FGF23 levels, and reducing body weight. The proresorptive effects were not observed in neutralizing anti-RANKL antibody-treated mice or Ob-VDR-cKO mice. Concomitantly, the hypercalcemic and weight loss effects of 1α,25(OH)_2_D_3_ were attenuated in both anti-RANKL antibody-treated mice and Ob-VDR-cKO mice. Taken together, our study suggests that in vivo administration of a toxic dose of 1α,25(OH)_2_D_3_ promotes bone resorption via VDR in osteoblast-lineage cells. We also discuss the physiological roles of VDR in osteoblast-lineage cells.

## Materials and Methods

### Animals

C57BL/6 mice were purchased from Japan SLC (Shizuoka, Japan). Osx-Cre^Tg/0^ (C57BL/6 genetic background) were purchased from the Jackson Laboratory (Bar Harbor, ME) (31). VDR-floxed mice (C57BL/6 genetic background) were generated by the authors (Y.Y., Y.N., and S.K.) (32). Mice were fed a standard diet containing 0.8% Ca and 0.65% phosphorus, and 2.3 IU/g vitamin D_3_ (Hi-Durability IRRD M/R, LabDiet, St. Louis, MO). All mice were housed in a specific pathogen-free facility at Matsumoto Dental University at 24 °C ± 2 °C and 50% to 60% humidity with a 12-hour light/dark cycle, and they were provided with sterilized water and diet ad libitum. All animal studies were reviewed and approved by the Research Animal Care and Use Committee of Matsumoto Dental University.

### Administration of drugs

A stock solution of 1α,25(OH)_2_D_3_ (Fujifilm Wako, Osaka, Japan) dissolved in ethanol was diluted in propylene glycol at a 1:9 ratio. 1α,25(OH)_2_D_3_ (0, 1, 5, 10, or 20 µg/kg of body weight/day) was administered daily for 4 days subcutaneously to 8-week-old male C57BL/6 mice. Anti-RANKL antibody (catalog No. 47104001, clone OYC1, Oriental Yeast, Tokyo, Japan) (33, 34) or control IgG (R&D systems, Minneapolis, MN) (5 mg/kg) was subcutaneously injected once to 8-week-old male C57BL/6 mice 4 days prior to 1α,25(OH)_2_D_3_ or vehicle treatment for 4 days. Eight-week-old Ob-VDR-cKO (Osx-Cre^Tg/0^; VDR^fl/fl^) or control (Osx-Cre^Tg/0^; VDR^+/+^) mice were treated with 1α,25(OH)_2_D_3_ (5 µg/kg of body weight/day) or vehicle for 4 days. In this Ob-VDR-cKO mouse experiment, we used both sexes, because the regulation of calcium-phosphate homeostasis and bone metabolism by 1α,25(OH)_2_D_3_ does not appear to be gender-dependent (1, 2). Mice were euthanized with ether 24 hours after the last 1α,25(OH)_2_D_3_ injection, and blood and tissue samples were collected. Mice were treated according to the institutional (Matsumoto Dental University) ethical guidelines for animal experimentation and safety.

### Serum biochemistry

Ca and phosphorus concentrations in serum were measured by the Calcium E-test kit (Fujifilm Wako) and the Phospha-C test kit (Fujifilm Wako), respectively. Serum C-terminal crosslinked telopeptide of type I collagen (CTX-I) concentrations were measured using the RatLaps EIA kit (catalog No. AC-06F1, Immunodiagnostic Systems, Boldon, UK) (35). Intact FGF23 levels were measured using the FGF-23 ELISA kit (catalog No. CY-4000, Kainos Laboratories, Tokyo, Japan) (36).

### Tartrate-resistant acid phosphatase staining

Femora were fixed with 4% (weight/volume) paraformaldehyde and decalciﬁed in 10% (weight/volume) EDTA (pH 7.3) for 3 weeks at 4 °C. Then, the specimens were dehydrated in a graded series of ethanol solutions, embedded in paraffin, and cut into 4-µm-thick sections. Double staining for methyl green and tartrate-resistant acid phosphatase (TRAP) was performed. TRAP-positive osteoclasts were detected as described previously (37). The number of osteoclasts per millimeter of trabecular bone surface was measured using Image J software (National Institutes of Health, Bethesda, MD). Images were obtained using Plan-Neofluar 5×/0.15 and Plan-Neofluar 40×/0.75 objectives (Carl Zeiss, Oberkochen, Germany) on a microscope (Axioplan 2 imaging; Carl Zeiss) with a digital camera (AxioCamHRc, Carl Zeiss). Images were captured using AxioVision software (Carl Zeiss). Figures were constructed using Photoshop (Adobe, San Jose, CA).

### Real-time reverse transcriptase–polymerase chain reaction

Tissue samples were collected, immediately soaked in TRIzol (Thermo Fisher Scientific, Waltham, MA), and homogenized with TissueLyser II (Qiagen, Hilden, Germany). Total RNA was extracted using the Purelink RNA mini kit (Thermo Fisher Scientific). First-strand cDNA was synthesized from total RNA with the oligo (dT)_12–18_ primer (Thermo Fisher Scientific) and ReverTra Ace reverse transcriptase (ToYoBo, Osaka) according to the manufacturer’s protocols. For preparation of bone samples, tibiae were isolated, and the epiphysis and adherent soft tissues were cut away and rubbed off with Kimwipe papers. The cleaned tibiae containing bone marrow were subjected to total RNA extraction. Real-time reverse transcriptase–polymerase chain reaction (RT-PCR) for the quantification of mRNA expression was performed using the Fast SYBR Green and StepOnePlus System (Thermo Fisher Scientific). The following temperature profile was used: 95 ^o^C for 20 seconds, followed by 40 cycles of 95 °C for 3 seconds and 60 °C for 30 seconds. Each gene expression level was calculated using a relative standard curve. Gene expression was normalized to that of glyceraldehyde 3-phosphate dehydrogenase (Gapdh). Mouse primers for Rankl, Opg, Ctsk, Cyp24a1, and Gapdh (Hokkaido System Science, Sapporo, Japan) are listed in Table 1.

**Table 1.** Primers Used in Real-time RT-PCR

Gene	Forward	Reverse
Gapdh	5´-TGTGTCCGTCGTGGATCTGA-3´	5´-TTGCTGTTGAAGTCGCAGGAG-3´
Rankl	5´-CATGTGCCACTGAGAACCTTGAA-3´	5´-CAGGTCCCAGCGCAATGTAAC-3´
Opg	5´-CATGAGGTTCCTGCACAGCTTC-3´	5´-ACAGCCCAGTGACCATTCCTAGTTA-3´
Ctsk	5´-CAGCAGAACGGAGGCATTGA-3´	5´-CTTTGCCGTGGCGTTATACATACA-3´
Cyp24a1	5´-CTGCCCATTGCGTTCTGT-3´	5´-TCTTGATTTGGGGGTGAAAA-3´

### Statistical analysis

**Figure 1.**
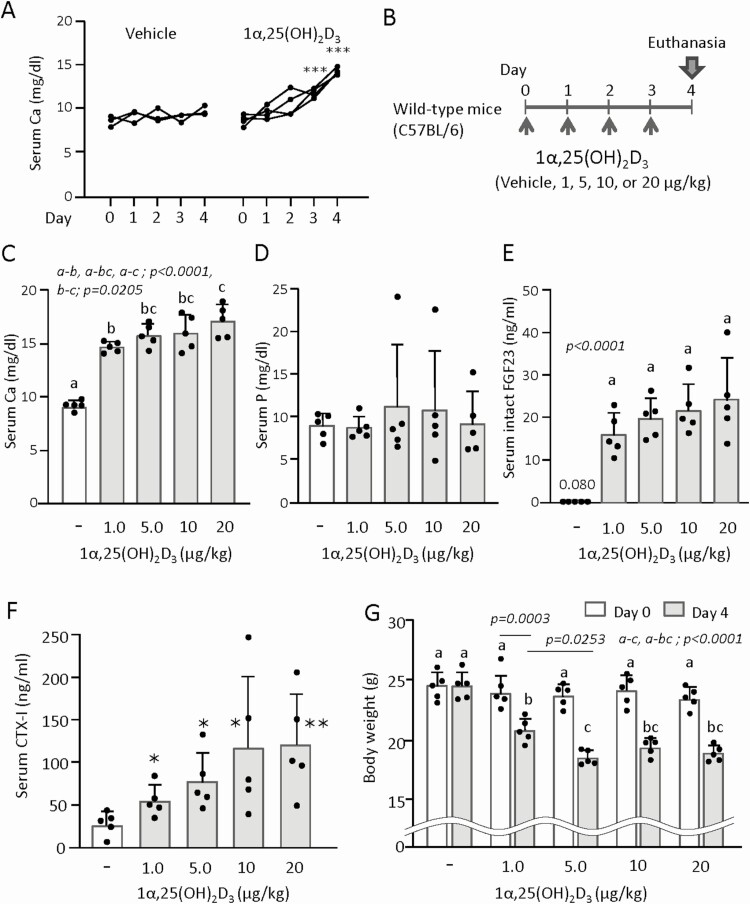
Dose-response effects of 1α,25(OH)_2_D_3_ on serum parameters. (A) Time-course changes of serum calcium (Ca) levels in a pilot test at a dosage of 1 μg/kg of body weight/day. The vehicle-treated group consisted of 1 male and 2 female mice. The 1α,25(OH)2D3-treated group consisted of 1 female and 3 male mice. (B) The experimental schedule for the determination of an optimal dose. Eight-week-old male C57BL/6 mice were administered different doses of 1α,25(OH)_2_D_3_ (1, 5, 10, or 20 μg/kg of body weight/day) daily for 4 days. Mice were euthanized at day 4 to collect blood samples, and then subjected to measurements of serum Ca (C), phosphorus (P) (D), intact FGF23 (E), CTX-I (F) levels, and body weight (G). Values represent the mean ± SD. *P < 0.05, **P < 0.01, ***P < 0.001; by 2-tailed Student *t* test; compared with the vehicle-treated (-) group. Groups sharing a letter are not significantly different (*P *> 0.05, 1-way ANOVA with post hoc Tukey test). b-bc, c-bc; not significant. Groups having different letters are significantly different (*P *< 0.05).

The statistical analysis was performed using the GraphPad Prism 8 statistical software (GraphPad Software, San Diego, CA). All data except for Fig. 1A are presented as the mean ± standard deviation (SD) of at least 4 mice. To compare 2 groups with normal distribution and equal variances, 2-tailed Student *t* test was used to assess significance. For analysis of 2 groups whose datasets do not have normal distribution or equal variances, Mann-Whitney *U* test was used. To compare multiple groups, 1-way analysis of variance (ANOVA) with the Tukey post hoc test was performed. A *P* value of < 0.05 was considered significant.

## Results

A pilot time-course experiment was performed to identify the optimal duration of 1α,25(OH)_2_D_3_ administration for the induction of hypercalcemia. 1α,25(OH)_2_D_3_ was injected at a daily dosage of 1 μg/kg of body weight to male and female C57BL/6 mice, whose sera were collected from tail vein at day 0, 1, 2, and 3, and by heart puncture at day 4 under ether anesthesia. At day 4, the mice injected with 1α,25(OH)_2_D_3_ were euthanized, because they looked exhausted. Daily injection of 1α,25(OH)_2_D_3_ caused daily increases of serum Ca levels throughout the 4-day follow-up period (Fig. 1A). To determine the in vivo optimal dose of 1α,25(OH)_2_D_3_ for the induction of bone resorption, 8-week-old male C57BL/6 mice were injected daily with different amounts of 1α,25(OH)_2_D_3_ (1, 5, 10, or 20 μg/kg of body weight/day) for 4 days and their sera were collected (Fig. 1B). Injection of 1α,25(OH)_2_D_3_ dose-dependently increased serum Ca levels (Fig. 1C). It did not significantly increase serum phosphorus levels, which varied greatly in each data set. Of note, 1 mouse in each group treated with ≥5 μg/kg of body weight/day exhibited severe hyperphosphatemia (>15 mg/dL) (Fig. 1D). Serum levels of intact FGF23, an osteocyte and osteoblast-derived key hormone in the calcium-phosphate homeostasis (7, 8, 38, 39), were drastically increased by 1α,25(OH)_2_D_3_ injection (Fig. 1E). Serum concentrations of C-terminal telopeptide of type I collagen (CTX-I), a bone resorption marker, were also upregulated by injection of 1α,25(OH)_2_D_3_ in a dose-dependent manner (Fig. 1F). The 1α,25(OH)_2_D_3_ injection dose-dependently reduced body weight (Fig. 1G). These results suggested that the administration of large amounts of 1α,25(OH)_2_D_3_ is toxic and stimulates bone resorption. One mouse died in the 10 μg/kg and in the 20 μg/kg of body weight/day group during the experiment. These mice were not included in Fig. 1. Therefore, we selected 5 μg/kg of body weight/day for 4 days as the optimal dose and period for further analyses of the proresorptive (i.e., toxic) action.

**Figure 2.**
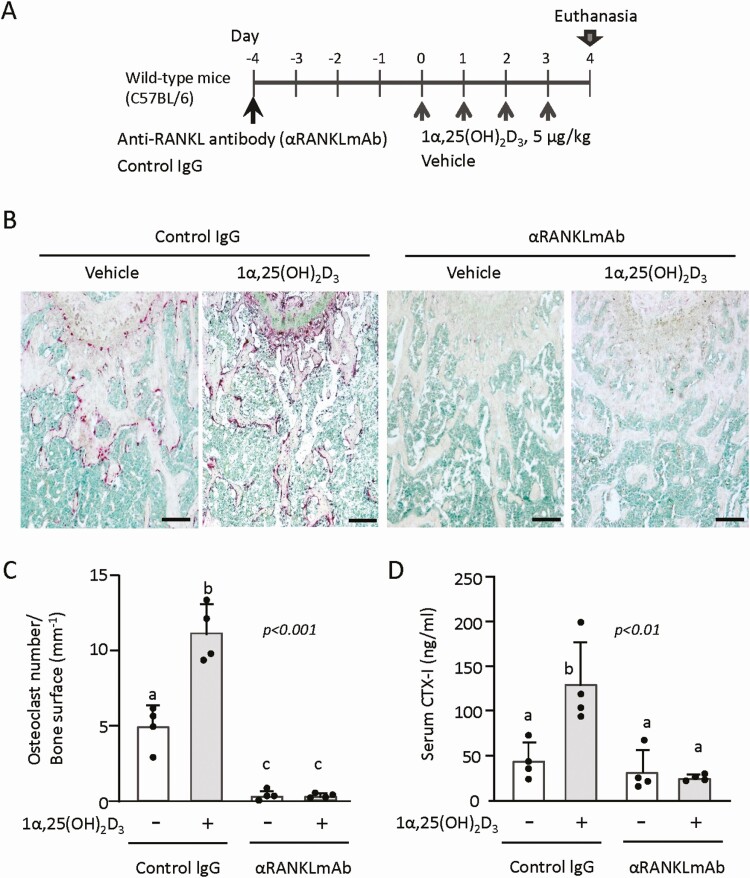
Effects of pretreatment with anti-RANKL monoclonal antibody (αRANKLmAb) on 1α,25(OH)_2_D_3_-induced bone resorption. (A) The experimental schedule. Male 8-week-old C57BL/6 mice were first subjected to a single injection of anti-RANKL antibody or control IgG (5 mg/kg of body weight) and 4 days later, the mice were administered 1α,25(OH)_2_D_3_ daily for 4 days at a dose of 5 μg/kg of body weight/day. Then, mice were euthanized to collect femora, tibiae, kidneys, and blood. (B) Histochemical analysis of the distal femora. Sections were subjected to TRAP (red) and methyl green staining. Cells stained red are osteoclasts. Bar = 200 µm. (C) Osteoclast numbers. (D) Serum CTX-I levels. Values represent the mean ± SD. Groups sharing a letter are not significantly different (*P *> 0.05, 1-way ANOVA with post hoc Tukey test). Groups having different letters are significantly different (*P *< 0.05).

A single pre-injection of neutralizing anti-RANKL antibody (OYC1) (34) into mice was shown to consistently suppress bone resorption even after administration of parathyroid hormone daily for 2 weeks (33). When mice are pretreated with anti-RANKL antibody, no 1α,25(OH)_2_D_3_-induced bone resorption should be observed. Therefore, a single dose of anti-RANKL antibody or control IgG (5 mg/kg of body weight) was administered to male C57BL/6 mice, and 4 days later, 1α,25(OH)_2_D_3_ (5 μg/kg of body weight/day) was daily administered for 4 days. At day 4, femora, tibiae, kidneys, and blood were collected from these mice (Fig. 2A). In control IgG-pretreated mice, a number of TRAP-positive osteoclasts were observed along the bone surface (Fig. 2B and 2C). When 1α,25(OH)_2_D_3_ was administered to these mice, an increased number of osteoclasts was observed. Pretreatment with anti-RANKL antibody markedly reduced osteoclast numbers in the vehicle-treated mice (Fig. 2B and 2C). Administration of 1α,25(OH)_2_D_3_ hardly induced osteoclasts in bone tissues in anti-RANKL antibody-pretreated mice (Fig. 2B, C). Consistent with this, 1α,25(OH)_2_D_3_ significantly increased serum CTX-I levels in control IgG-pretreated mice, but not in anti-RANKL antibody-pretreated mice (Fig. 2D). These results suggested that a toxic dose of 1α,25(OH)_2_D_3_ can induce bone resorption in vivo.

**Figure 3.**
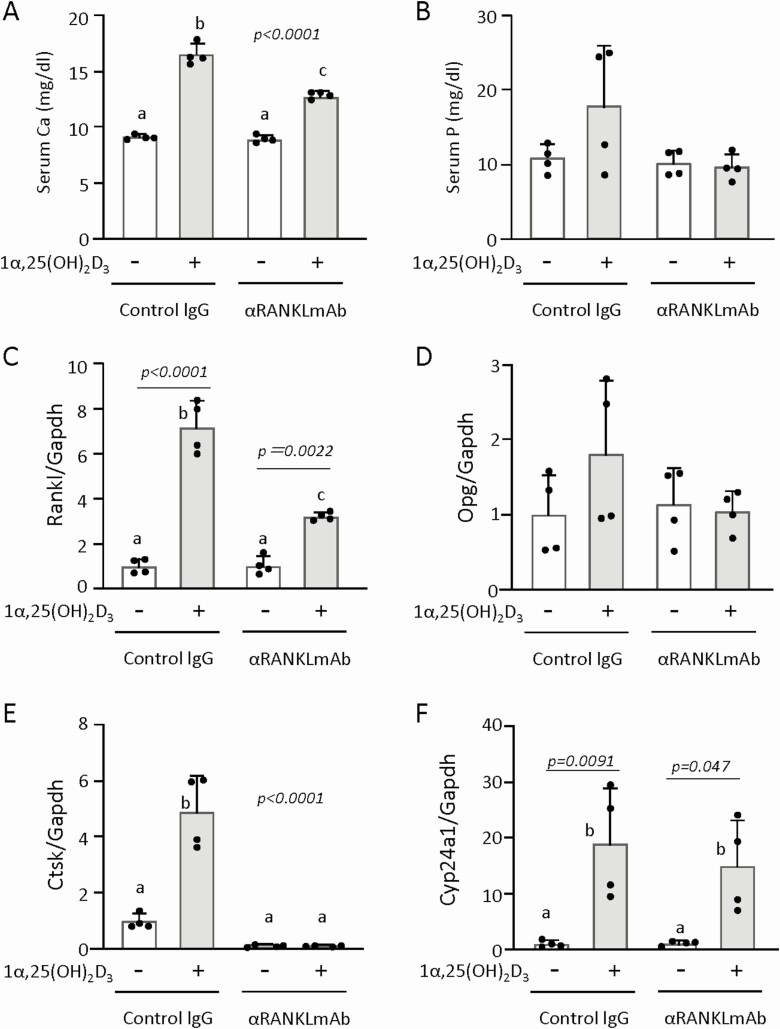
Effects of pretreatment with αRANKLmAb on 1α,25(OH)_2_D_3_-induced changes in serum Ca and phosphorus (P), and mRNA expression in bone and kidneys. Results were obtained from the same experiments as Fig. 2. Serum levels of Ca (A) and P (B) were measured. mRNA expression levels of RANKL (C), OPG (D), and Ctsk (E) in tibiae, and Cyp24A1 in kidneys (F) were quantified by real-time RT-PCR. Values represent the mean ± SD. Groups sharing a letter are not significantly different (*P *> 0.05, 1-way ANOVA with post hoc Tukey test). Groups having different letters are significantly different (*P *< 0.05).

Next, we compared serum biochemical parameters and mRNA expression in bone and kidneys between control IgG-pretreated and anti-RANKL antibody-pretreated mice. Administration of 1α,25(OH)_2_D_3_ significantly increased serum Ca levels in control IgG-pretreated mice. The 1α,25(OH)_2_D_3_ administration also increased serum Ca levels in anti-RANKL antibody-pretreated mice, but the extent of the increase was smaller (~50%) than that observed in control IgG-pretreated mice (Fig. 3A). Serum phosphorus levels were not significantly increased by 1α,25(OH)_2_D_3_ administration in control IgG-pretreated or anti-RANKL antibody-pretreated mice. Of note, the 2 of 4 1α,25(OH)_2_D_3_-treated mice in the control IgG-pretreated group exhibited severe hyperphosphatemia (>15 mg/dL), whereas 1α,25(OH)_2_D_3_ did not induce hyperphosphatemia in any of the anti-RANKL antibody-pretreated mice (Fig. 3B). Upregulation of RANKL mRNA expression by 1α,25(OH)_2_D_3_ in bone was observed in both control and anti-RANKL antibody-pretreated mice (Fig. 3C). Expression levels of OPG mRNA in bone were not altered by 1α,25(OH)_2_D_3_ (Fig. 3D). On the other hand, upregulated expression of cathepsin K (Ctsk) mRNA, a marker enzyme of osteoclasts, in bone was observed in control mice, but not in anti-RANKL antibody-pretreated mice (Fig. 3E). Cyp24A1, an enzyme that hydroxylates C-24 of 1α,25(OH)_2_D_3_ and 25(OH)D_3_, is induced by 1α,25(OH)_2_D_3_ in the target tissues through VDR (1, 2). The increase in renal Cyp24A1 expression by 1α,25(OH)_2_D_3_ was comparable between control mice and anti-RANKL pretreated mice (Fig. 3F). This suggested (i) that 1α,25(OH)_2_D_3_ stimulates osteoclastogenesis through the upregulation of RANKL expression in bone tissues, and (ii) that the inhibition of 1α,25(OH)_2_D_3_-induced osteoclastogenesis by anti-RANKL antibody can reduce the hypercalcemic effects of 1α,25(OH)_2_D_3_.

**Figure 4.**
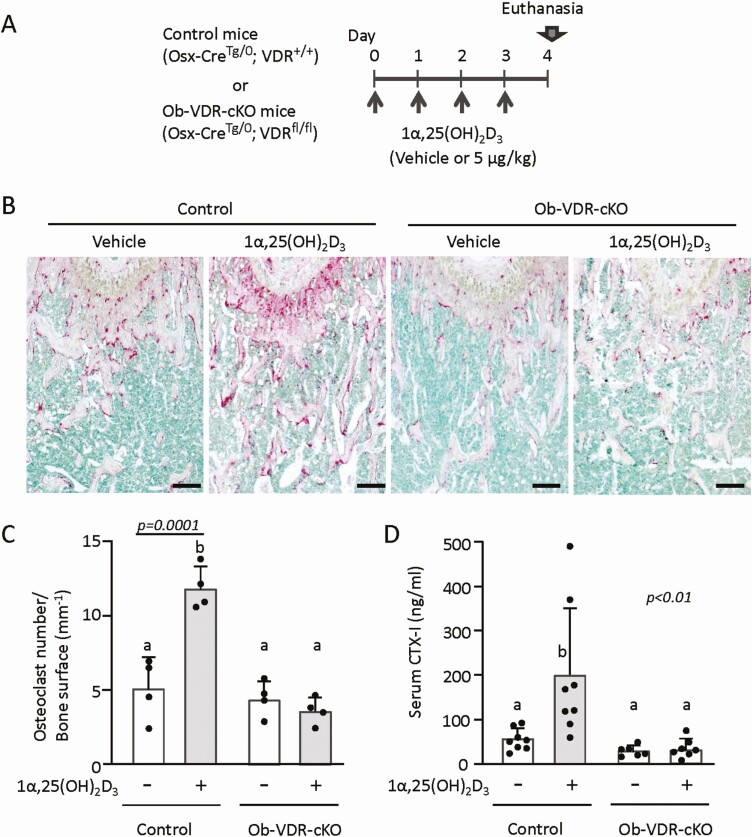
Effects of osteoblast-lineage-specific ablation of VDR on 1α,25(OH)_2_D_3_-induced bone resorption. (A) The experimental schedule: 8-week-old Ob-VDR-cKO mice (male: n = 5; female: n = 2) or control mice (male: n = 4) were administered 1α,25(OH)_2_D_3_ for 4 days at a dose of 5 μg/kg of body weight/day. For treatment with vehicle, 8-week-old Ob-VDR-cKO mice (male: n = 4; female: n = 2) or control mice (male: n = 2; female: n = 2) were used. Then, mice were euthanized to collect femora, tibiae, kidneys, and blood. (B) Histochemical analysis of the distal femora. Sections were subjected to TRAP (red) and methyl green staining. Cells stained red are osteoclasts. Bar = 200 µm. (C) Osteoclast numbers. (D) Serum CTX-I levels. Values represent the mean ± SD. Groups sharing a letter are not significantly different (*P *> 0.05, 1-way ANOVA with post hoc Tukey test). Groups having different letters are significantly different (*P *< 0.05).

**Figure 5.**
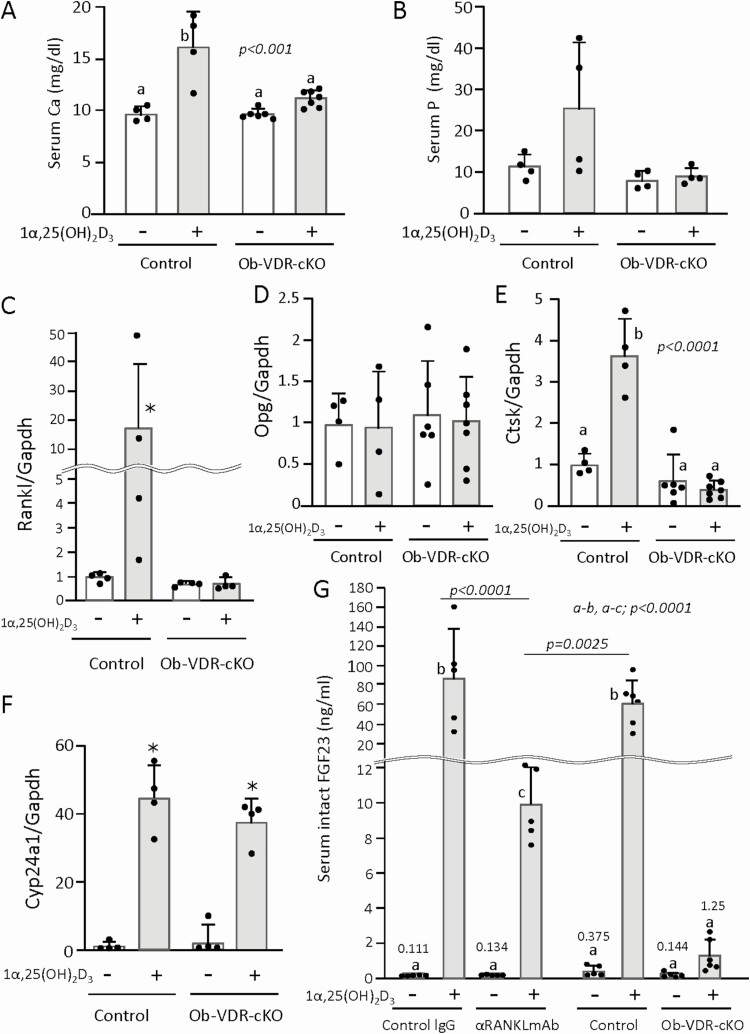
Effects of osteoblast-lineage-specific ablation of VDR on 1α,25(OH)_2_D_3_-induced changes in serum Ca and P, and mRNA expression in bone and kidneys. Results were obtained from the same experiments as Fig. 4. Serum levels of Ca (A) and P (B) and intact FGF23 (G) were measured. mRNA expression levels of RANKL (C), OPG (D), and Ctsk (E) in tibiae, and Cyp24A1 in kidneys (F) were quantified by real-time RT-PCR. Results of serum intact FGF23 levels (G) obtained from the same experiments as Fig. 2 were also shown for a close comparison between the effects of pretreatment with αRANKLmAb and those of osteoblast-lineage-specific ablation of VDR. Values represent the mean ± SD. *P < 0.05; by 2-tailed Mann-Whitney *U* test; compared with the vehicle-treated (-) group. Groups sharing a letter are not significantly different (*P *> 0.05, 1-way ANOVA with post hoc Tukey test). Groups having different letters are significantly different (*P *< 0.05).

To examine whether the proresorptive effects of a toxic dose of 1α,25(OH)_2_D_3_ were mediated by VDR in osteoblast-lineage cells, we evaluated bone resorption in 1α,25(OH)_2_D_3_-treated Ob-VDR-cKO mice. 1α,25(OH)_2_D_3_ (5 μg/kg of body weight/day) was administered daily for 4 days to control (Osx-Cre^Tg/0^, VDR^+/+^) mice and Ob-VDR-cKO (Osx-Cre ^Tg/0^;VDR ^fl/fl^) mice of both sexes, and their femora, tibiae, and blood were collected (Fig. 4A). TRAP-positive osteoclasts were similarly observed in both control and Ob-VDR-cKO mice treated with the vehicle (Fig. 4B, C). Administration of 1α,25(OH)_2_D_3_ markedly increased the number of TRAP-positive osteoclasts in the femora of control mice, but not in those of Ob-VDR-cKO mice (Fig. 4B, C). Injection of 1α,25(OH)_2_D_3_ increased serum CTX-I levels in control mice, but not in Ob-VDR-cKO mice (Fig. 4D). Therefore, osteoclast formation induced by 1α,25(OH)_2_D_3_ was mediated by VDR in osteoblast-lineage cells. We then analyzed serum biochemical parameters and mRNA expression of genes related to osteoclastogenesis in bone tissues of control and Ob-VDR-cKO mice. Administration of 1α,25(OH)_2_D_3_ significantly increased serum Ca levels in control mice, but not in Ob-VDR-cKO mice (Fig. 5A). Serum phosphorus levels were not significantly increased by 1α,25(OH)_2_D_3_ in both control and Ob-VDR-cKO mice. Of note, 2 of 4 1α,25(OH)_2_D_3_ -treated control mice exhibited severe hyperphosphatemia (>30 mg/dL), whereas 1α,25(OH)_2_D_3_ did not induce hyperphosphatemia in any of the Ob-VDR-cKO mice (Fig. 5B). Upregulation of RANKL mRNA expression by 1α,25(OH)_2_D_3_ was observed in bone of control mice, but not in bone of Ob-VDR-cKO mice (Fig. 5C). Expression levels of OPG mRNA in bone were not altered by 1α,25(OH)_2_D_3_ (Fig. 5D). Consistent with the changes in osteoclast numbers and RANKL expression, upregulated expression of Ctsk mRNA by 1α,25(OH)_2_D_3_ in bone was observed in control mice, but not in Ob-VDR-cKO mice (Fig. 5E). Expression of renal Cyp24A1 mRNA was increased by 1α,25(OH)_2_D_3_ similarly in both control and Ob-VDR-cKO mice (Fig. 5F). These observations suggested that in vivo administration of a toxic dose of 1α,25(OH)_2_D_3_ can induce osteoclast formation through VDR-mediated control of gene expression in osteoblast-lineage cells. Serum levels of intact FGF23 were several-hundred-fold increased by 1α,25(OH)_2_D_3_ administration in the control (control IgG and control) groups. 1α,25(OH)_2_D_3_ increased serum FGF23 levels a hundred times in anti-RANKL antibody-pretreated mice, although the pretreatment with anti-RANKL antibody attenuated the increase of FGF23 levels induced by 1α,25(OH)_2_D_3_ administration. Notably, 1α,25(OH)_2_D_3_ failed to significantly increase serum FGF23 levels in Ob-VDR-cKO mice, indicating that production of FGF23 is tightly regulated by VDR in osteoblast-lineage cells (Fig. 5G).

**Figure 6.**
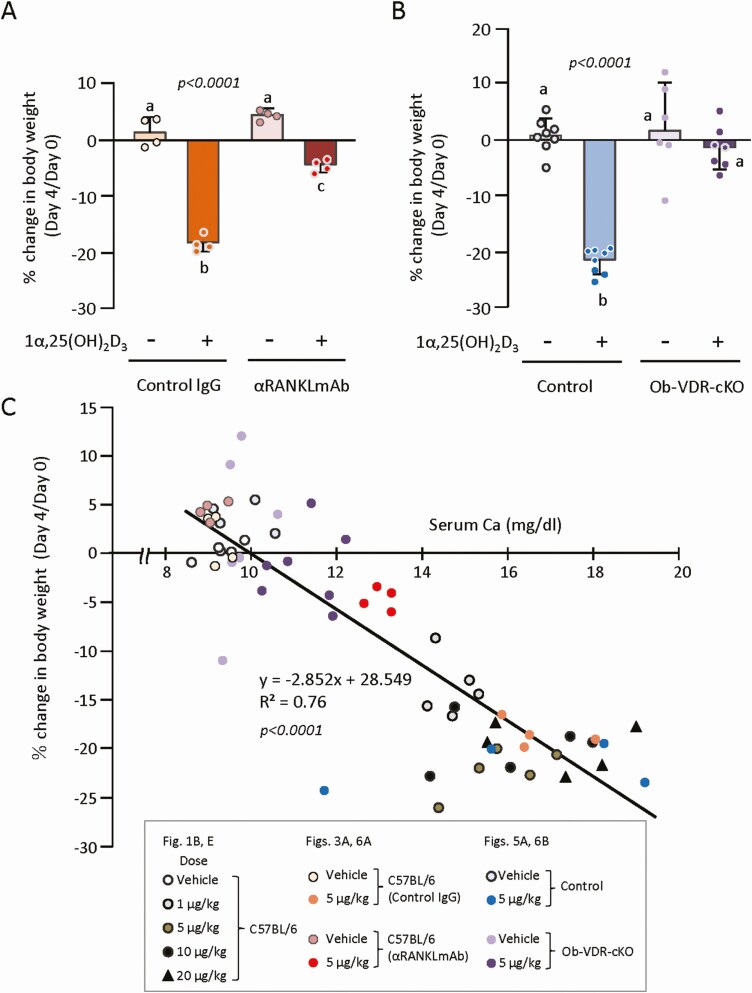
Effects of pretreatment with αRANKLmAb and osteoblast-lineage-specific ablation of VDR on 1α,25(OH)_2_D_3_-induced body weight loss. Results were obtained from the same experiments as Figs. 2 and 4. Body weight was measured on day 0 and day 4 in control IgG and αRANKLmAb-pretreated mice (A); in control and Ob-VDR-cKO mice (B). The % change in body weight was calculated in each mouse. Values represent the mean ± SD. Groups sharing a letter are not significantly different (*P *> 0.05, 1-way ANOVA with post hoc Tukey’s test). Groups having different letters are significantly different (*P *< 0.05). (C) A scatter plot with a simple regression line illustrating the relationship between serum Ca levels and the % change in body weight. All data of the 2 parameters in this study are plotted on the graph.

Lastly, we assessed the relationship between body weight loss and bone resorption induced by 1α,25(OH)_2_D_3_. Administration of 1α,25(OH)_2_D_3_ (5 μg/kg of body weight/day for 4 days) significantly reduced the body weight in control IgG-pretreated mice and anti-RANKL antibody-pretreated mice. The reduction induced by 1α,25(OH)_2_D_3_ was greatly attenuated in anti-RANKL antibody-pretreated mice (Fig. 6A). Administration of 1α,25(OH)_2_D_3_ significantly reduced the body weight in control mice, but not in Ob-VDR-cKO mice (Fig. 6B). To take a closer look at the relationship between the % changes in body weight and serum Ca levels, all data of these parameters in this study were subjected to a correlation plot analysis (Fig. 6C). The correlation squared (R^2^) was 0.76, and the slope was −2.85 (*P *< 0.0001), indicating that the body weight loss was sufficiently explained by hypercalcemia. These results suggested that inhibition of bone resorption can prevent 1α,25(OH)_2_D_3_ toxicity.

## Discussion

**Figure 7.**
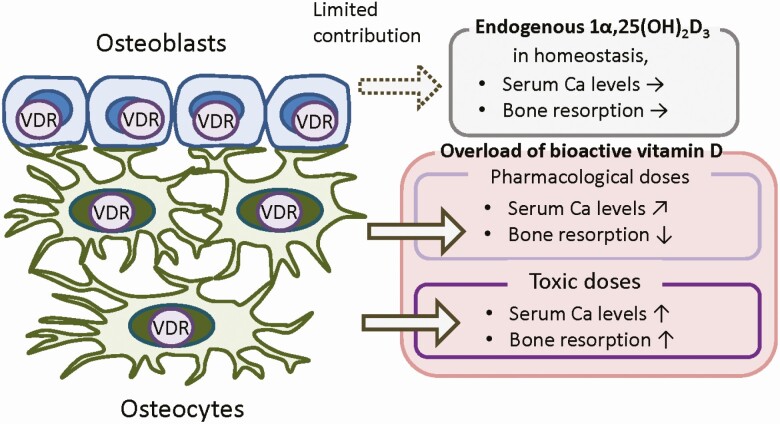
Roles of VDR in osteoblast-lineage cells (osteoblasts and osteocytes) deduced from our studies. Our current and previous studies revealed that VDR in osteoblast-lineage cells is dispensable for regulation of serum Ca levels and bone resorption in homeostasis. VDR in osteoblast-lineage cells is involved in regulation of serum Ca levels and bone resorption specifically when bioactive vitamin D is overloaded. In previous studies, we demonstrated that long-term administration of a pharmacological dose of a 1α,25(OH)_2_D_3_ analog, induces modest increases of serum Ca levels and suppression of bone resorption via VDR in osteoblast-lineage cells. In this study, we demonstrated that toxic doses of 1α,25(OH)_2_D_3_ increase serum Ca levels and bone resorption via VDR in osteoblast-lineage cells.

We demonstrated (i) that high-dose administration of 1α,25(OH)_2_D_3_ can stimulate bone resorption and (ii) that this stimulation is due to VDR in osteoblast-lineage cells. Administration of 1α,25(OH)_2_D_3_ (5 μg/kg of body weight/day for 4 days) to control mice increased osteoclast numbers, serum Ca, serum intact FGF23, and serum CTX-I levels. When 1α,25(OH)_2_D_3_ was administered to mice pretreated with anti-RANKL antibody, the 1α,25(OH)_2_D_3_-induced bone resorption was suppressed and the increase in serum Ca was attenuated. Ob-VDR-cKO mice did not exhibit the 1α,25(OH)_2_D_3_-induced increase in these bone resorption-related parameters. A high variability in serum phosphorus concentrations induced by 1α,25(OH)_2_D_3_ administration was probably due to osteoclastic bone resorption, which enhanced release of phosphate and Ca from bone. Subsequently, enhancement of urinary phosphate excretion likely occurred due to 1α,25(OH)_2_D_3_-induced increase in serum intact FGF23 levels. FGF23 has been discovered to be a phosphaturic hormone that suppresses phosphate reabsorption in renal proximal tubules (40, 41). Simultaneous enhancement of bone resorption and FGF23 production seemed to fluctuate serum phosphorus levels. This fluctuation was not observed in anti-RANKL antibody-pretreated mice or Ob-VDR-cKO mice. The present study revealed that a toxic dose of 1α,25(OH)_2_D_3_ promotes bone resorption and this toxic action is mediated by VDR in osteoblast-lineage cells in vivo. It is well established that 1α,25(OH)_2_D_3_ can stimulate osteoclast formation in coculture of mouse osteoblastic cells and hematopoietic cells through VDR expressed in osteoblastic cells (42). Taken together, we conclude that 1α,25(OH)_2_D_3_ plays a proresorptive role via VDR in osteoblast-lineage cells in in vitro and in vivo (Fig. 7).

Long-term administration of a pharmacological dose of eldecalcitol as well as alfacalcidol suppressed bone resorption and increased bone mass (17-23, 28-30). Transgenically overexpressed VDR in mature osteoblast-lineage cells in mice also increased bone mass by suppressing bone resorption (24, 25). These antiresorptive effects were not observed in Ob-VDR-cKO mice (30). Thus, the proresorptive action and the antiresorptive action of bioactive vitamin D are mediated by VDR in osteoblast-lineage cells. How does VDR in osteoblast-lineage cells select the mode of action? Increases in bone resorption by 1α,25(OH)_2_D_3_ are shown to require a large dose and relatively short treatment period of around 4 days. In contrast, inhibition of bone resorption by bioactive vitamin D needs lower doses (=pharmacological doses) and a long treatment period of more than 4 weeks. This long and modest stimulation with eldecalcitol and stimulation by overexpressed VDR in mature osteoblast-lineage cells are shown to reduce the RANKL/OPG ratio in bone tissues (25, 29, 30). It is well known that the RANKL/OPG ratio is regulated by factors other than 1α,25(OH)_2_D_3_, such as parathyroid hormone and various cytokines (16). Long-term administration of a pharmacological dose of bioactive vitamin D may regulate the secretion and expression of such regulators of bone resorption via VDR in osteoblast-lineage cells.

Some studies suggested that vitamin D_3_ analogs selectively recruit VDR coactivators for transcription (43, 44). However, we saw just similar effects on bone resorption between eldecalcitol and 1α,25(OH)_2_D_3_ at a pharmacological dose (29) and also at a toxic dose of 5 μg/kg of body weight/day for 4 days (serum Ca: 9.3 ± 0.52 mg/dL [vehicle, n = 4], 16.3 ± 0.98^a^ mg/dL [eldecalcitol, n = 5], 15.4 ± 0.91^a^ mg/dL [1α,25(OH)_2_D_3_, n = 4]), ^a^*P *< 0.0001; serum CTX-I: 17.9 ± 5.5 ng/ml [vehicle, n = 4], 146 ± 59.7^a^ ng/mL (eldecalcitol, n = 5), 110 ± 44.3^a^ ng/mL [1α,25(OH)_2_D_3_, n = 4], ^a^*P *< 0.05, 1-way ANOVA with Tukey test). Coactivator complexes recruited by toxic doses of 1α,25(OH)_2_D_3_ and eldecalcitol may be different from those by pharmacological doses and this is one of the possibilities for the opposite actions. Elucidation of the mechanism by which VDR in osteoblast-lineage cells exert biphasic and opposite effects on bone resorption currently remains as an unresolved and valuable challenge (Fig. 7).

Bioactive vitamin D regulates serum Ca levels. Does VDR in osteoblast-lineage cells play important roles in this central function in physiology? Vitamin D-deficient/insufficient animals and humans, as well as global VDR-KO mice, exhibit hypocalcemia and rickets/osteomalacia (2, 45-47). We previously analyzed VDR expression in bone tissues by immunohistochemistry and found that VDR was expressed exclusively in osteoblasts and osteocytes among bone cells, and was limited in chondrocytes, osteoclasts, and bone marrow cells (30). Although VDR expression was almost completely diminished in bone of Ob-VDR-cKO mice, no notable abnormalities were found in bone formation, bone resorption, serum Ca levels, or overall health of these mice in homeostasis (30). Late-osteoblast-lineage (mature osteoblast and osteocyte)-specific VDR-cKO mice also showed no abnormalities in the bone phenotype, and serum Ca levels (48). Therefore, 1α,25(OH)_2_D_3_-VDR signaling in extraskeletal tissues is essential for Ca homeostasis. Global VDR-KO mice do not exhibit abnormalities before weaning (3 weeks of age) and develop rickets or osteomalacia after weaning (47). A high-Ca diet can cure rickets/osteomalacia in global VDR-KO mice (49, 50). Transgenic expression of VDR in the intestine in global VDR-KO mice normalized intestinal Ca absorption and mineralization of bone (51). These findings suggest that 1α,25(OH)_2_D_3_ has evolved as a hormone to stimulate Ca absorption from the intestine. 1α,25(OH)_2_D_3_ increases reabsorption of Ca in distal tubules in kidneys (50, 52, 53). In global VDR-KO mice, renal Ca reabsorption is suppressed, and consequently, their Ca excretion to urine is increased (50). Thus, bioactive vitamin D also regulates renal Ca reabsorption. VDR in osteoblast-lineage cells was unlikely involved in such renal and intestinal regulation of serum Ca levels in homeostasis (Fig. 7). Long-term administration of a pharmacological dose of eldecalcitol to mice slightly but significantly increased serum Ca levels (29, 30). This mild hypercalcemic effect of eldecalcitol was also not observed in Ob-VDR-cKO mice (30). Thus, VDR in osteoblast-lineage cells may function for regulation of serum Ca levels and bone resorption specifically when bioactive vitamin D is overloaded by administration (Fig. 7).

1α,25(OH)_2_D_3_ did not enhance bone resorption in mice pretreated with anti-RANKL antibody, as that was assessed by osteoclast numbers and CTX-I. However, 1α,25(OH)_2_D_3_ increased serum Ca levels in these mice but not in Ob-VDR-cKO mice. What is the reason for this? If this hypercalcemic action was caused by effects of 1α,25(OH)_2_D_3_ on intestine or kidney, it was surprising that 1α,25(OH)_2_D_3_ did not increase serum Ca levels in Ob-VDR-cKO mice. We consider that the difference in 1α,25(OH)_2_D_3_-induced FGF23 production between anti-RANKL antibody-pretreated mice and Ob-VDR-cKO is the reason for the presence or absence of the hypercalcemic action. FGF23 is recently reported to promote Ca reabsorption in renal distal tubules through the transient receptor potential cation channel subfamily V member 5 (TRPV5) in a VDR-independent manner (38). FGF23/VDR-double KO mice on rescue diet showed more severe renal Ca wasting than VDR-KO mice on rescue diet (38). Consistently, mice with conditional deletion of FGF receptor 1 (a receptor for FGF23) in the distal tubule exhibit renal Ca wasting and reduced trabecular bone mineral density (54). Therefore, we speculate that sufficiently increased FGF23 by 1α,25(OH)_2_D_3_ administration enhanced renal Ca reabsorption through FGF receptor 1 in distal tubules and contributed to the increase of serum Ca levels in control mice and anti-RANKL-pretreated mice.

Then, the result of FGF23 levels raises another question about smaller increases in serum FGF23 levels by 1α,25(OH)_2_D_3_ administration in anti-RANKL-pretreated mice than in control IgG-pretreated mice. Quinn et al (39) reported that not only high serum phosphorus levels but also high serum Ca levels increased FGF23 production in bone. However, the best correlation between serum Ca and phosphorus and serum FGF23 levels was found between serum FGF23 levels and the Ca × phosphorus product. This means FGF23 synthesis is highly interrelated to both serum phosphorus and Ca levels. The relationship between the Ca × phosphorus product and serum FGF23 levels was not linear but quadratic. This quadratic relationship is likely to be the reason for the smaller increases in FGF23 levels in anti-RANKL-pretreated mice, as the extent of increases in serum Ca levels by 1α,25(OH)_2_D_3_ administration was smaller in anti-RANKL-pretreated mice than in control IgG-pretreated mice.

Daily administration of a toxic dose of 1α,25(OH)_2_D_3_ reduced the body weight proportionally to the increase of serum Ca levels. In addition, 1α,25(OH)_2_D_3_ administration suppressed locomotor activity of the mice; however, the body weight loss and decrease in locomotor activity by 1α,25(OH)_2_D_3_ were absent in anti-RANKL antibody-pretreated mice and Ob-VDR-cKO mice. This suggests that bone resorption is related to vitamin D toxicity (hypervitaminosis D). We are currently investigating the relationship between the inhibition of bone resorption and hypervitaminosis D using anti-RANKL antibody and Ob-VDR-cKO mice. Future studies are expected to reveal new roles of VDR in osteoblast-lineage cells and the mechanism by which inactivation of VDR signaling in osteoblast-lineage cells prevents the toxic action of vitamin D.

## Data Availability

All data generated or analyzed during this study are included in this published article or in the data repositories listed in References.

## Abbreviations

1α,25(OH)_2_D_3_: 1α,25-dihydroxyvitamin D_3_
25(OH)D_3_: 25-hydroxyvitamin D_3_
ANOVA: analysis of variance
Ca: calcium
Ctsk: cathepsin K
CTX-I: C-terminal crosslinked telopeptide of type I collagen
Cyp24a1: cytochrome P450 family 24 subfamily A member 1
FGF23: fibroblast growth factor 23
Gapdh: glyceraldehyde 3-phosphate dehydrogenase
Ob-VDR-cKO: osteoblast-lineage-specific VDR conditional knockout
OPG: osteoprotegerin
Osx: osterix
RANKL: receptor activator of NF-κB ligand
RT-PCR: reverse transcriptase–polymerase chain reaction
TRAP: tartrate-resistant acid phosphatase
VDR: vitamin D receptor

## Additional Information

***Disclosure Summary*:** The authors have nothing to disclose.
